# YF-H-2015005, a CXCR4 Antagonist, for the Mobilization of Hematopoietic Stem Cells in Non-Hodgkin Lymphoma Patients: A Randomized, Controlled, Phase 3 Clinical Trial

**DOI:** 10.3389/fmed.2021.609116

**Published:** 2021-02-02

**Authors:** Weiping Liu, Yufu Li, Quanshun Wang, Hang Su, Kaiyang Ding, Yuerong Shuang, Sujun Gao, Dehui Zou, Hongmei Jing, Ye Chai, Yicheng Zhang, Lihong Liu, Chunling Wang, Hui Liu, Jinying Lin, Haiyan Zhu, Chen Yao, Xiaoyan Yan, Meixia Shang, Shufang Wang, Fengyuan Chang, Xiaopei Wang, Jun Zhu, Yuqin Song

**Affiliations:** ^1^Key Laboratory of Carcinogenesis and Translational Research (Ministry of Education), Department of Lymphoma, Peking University Cancer Hospital & Institute, Beijing, China; ^2^Department of Hematology, Henan Cancer Hospital, Zhengzhou, China; ^3^Department of Hematology, The First Medical Center, Chinese PLA General Hospital, Beijing, China; ^4^Department of Lymphoma, The Fifth Medical Center, Chinese PLA General Hospital, Beijing, China; ^5^Department of Hematology, Anhui Provincial Cancer Hospital, Hefei, China; ^6^Department of Lymphoma & Hematology, Jiangxi Cancer Hospital, Nanchang, China; ^7^Department of Hematology, First Affiliated Hospital of Jilin University, Changchun, China; ^8^Department of Hematology, Chinese Academy of Medical Sciences & Peking Union Medical College, Tianjin, China; ^9^Department of Hematology, Peking University Third Hospital, Beijing, China; ^10^Department of Hematology, Lanzhou University Second Hospital, Lanzhou, China; ^11^Department of Hematology, Tongji Medical College, Huazhong University of Science and Technology, Wuhan, China; ^12^Department of Hematology, The Fourth Hospital of Hebei Medical University, Shijiazhuang, China; ^13^Department of Hematology, Huai'an First People's Hospital, Nanjing Medical University, Huai'an, China; ^14^Department of Hematology, Beijing Hospital, Beijing, China; ^15^Department of Hematology, People's Hospital of Guangxi Zhuang Autonomous Region, Nanning, China; ^16^Department of Medical Statistics, Peking University First Hospital, Beijing, China; ^17^Peking University Clinical Research Institute, Beijing, China; ^18^Hefei Yifan Biopharmaceuticals Inc., Economic Development Zone, Hefei, China

**Keywords:** hematopoietic stem cell mobilization, therapeutics, treatment outcome, safety, lymphoma, non-Hodgkin

## Abstract

**Background:** YF-H-2015005, a novel CXCR4 antagonist, has been proven to increase the quantities of circulating hematopoietic stem cells (HSCs), which results in an adequate collection of HSCs in non-Hodgkin lymphoma (NHL) patients.

**Methods:** This was a multicenter, double-blind, randomized (1:1), placebo-controlled phase III clinical trial. All patients received granulocyte colony-stimulating factor (G-CSF) for up to 8 consecutive days. YF-H-2015005 or placebo was administrated on the evening of day 4 and continued daily for up to 4 days. Apheresis was conducted 9–10 h after each dose of YF-H-2015005 or placebo. The primary endpoint was the proportion of NHL patients procuring ≥5 × 10^6^/kg CD34^+^ HSCs within ≤4 apheresis sessions.

**Results:** In total, 101 patients with NHL were enrolled. The proportions of patients achieving primary endpoint were 57 and 12% in YF-H-2015005 and placebo groups, respectively (*P* < 0.001). Moreover, a higher proportion of YF-H-2015005-treated patients reached a minimum target collection of ≥2 × 10^6^/kg CD34^+^ HSCs in ≤4 apheresis days compared to placebo-treated patients (86 vs. 38%, *P* < 0.001). Furthermore, the median time to collect ≥2 or 5 × 10^6^/kg CD34+ HSCs were 1 and 3 days in YF-H-2015005-treated patients, but 4 days and not reached in placebo-treated patients, respectively. No severe treatment emergent adverse events were observed in both YF-H-2015005 treatment and placebo groups.

**Conclusions:** YF-H-2015005 plus G-CSF regimen was a tolerable combination with high efficacy, which might be used to rapidly mobilize and collect HSCs in NHL patients.

## Introduction

Autologous hematopoietic stem cell transplantation (AHSCT) is often used as a consolidative therapy for patients with newly diagnosed non-Hodgkin lymphoma (NHL) or salvage therapy for those with relapsed or refractory disease (1–4). The mobilization and collection of adequate CD34^+^ hematopoietic stem cells (HSCs) is crucial for supporting AHSCT, of which granulocyte colony-stimulating factor (G-CSF) alone or G-CSF plus chemotherapy have been the most common mobilization methods (5–8). Moreover, plerixafor, a CXCR4 antagonist, has been demonstrated to optimize the mobilization procedures in patients with NHL and multiple myeloma, due to its capability to enhance the HSC mobilization effect of G-CSF (9–12).

YF-H-2015005, a biosimilar to plerixafor, has been found to increase the mean peripheral blood CD34+ cell counts by 2.0–2.9-fold and improve the quantity of mobilized CD34^+^ HSCs in peripheral blood with good tolerance, which can serve as a promising therapy for NHL patients undergoing AHSCT (13). The multicenter, randomized, double-blind, placebo-controlled, phase III study aimed to assess the effectiveness and tolerability of YF-H-2015005 plus G-CSF for mobilizing HSCs in NHL patients.

## Materials and Methods

### Study Design

This was a multi-center, double-blind, randomized, placebo-controlled phase 3 clinical trial, which was conducted in compliance with the Declaration of Helsinki. Ethical approval was obtained from the Ethics Committee at Peking University Cancer Hospital and Institute and the participating centers' institutional review boards. Written informed consent was signed by all patients prior to randomization. This trial was registered on www.chinadrugtrials.org.cn (Clinical trial registration number: CTR20161069).

The study consisted of two periods: (i) mobilization period was the time from random assignment to 24 h after the last apheresis and (ii) follow-up period was the time from 24 h after the last apheresis to 30 days after transfusion of HSCs for those patients who received transplantation or to 30 days after the last apheresis for those patients who did not receive transplantation.

### Patient Eligibility

The key inclusion criteria included NHL patients with eligible for AHSCT, aged 18–65 years, Eastern Cooperative Oncology Group (ECOG) performance status score ≤1, achieving complete or partial remission after first- or second-line therapy, and ≥4 weeks following the last cycle of chemotherapy. AHSCT was performed as consolidation therapy for mantle cell lymphoma, peripheral T/NK cell lymphoma, lymphoblastic lymphoma, and high-risk diffuse large B-cell lymphoma, while salvage therapy for relapsed/refractory NHL.

The key exclusion criteria included patients with chronic lymphocytic leukemia, previous HSC collection or transplantation, a history of pelvic radiotherapy, a history of radio-immunotherapy such as tositumomab and ibritumomab tiuxetan, treatment with carmustine or fludarabine within 6 weeks, receipt of G-CSF within 2 weeks or granulocyte-macrophage colony-stimulating factor within 3 weeks before randomization, and active bone marrow involvement before randomization.

### Treatment Protocol

All patients received G-CSF with a dose of 10 μg/kg per day in each morning for up to 8 consecutive days. Starting on the evening of day 4, they received either placebo or YF-H-2015005 (0.24 mg/kg) daily for up to 4 days. Circulating CD34+ cell numbers and complete blood cell count were monitored from day 4 to the last apheresis day.

Apheresis was performed by standard methods (at least 3 times blood volume), which was initiated on day 5 after the morning dose of G-CSF and continued daily for up to 4 days or until ≥5 × 10^6^/kg CD34^+^ HSCs. Assessment of CD34^+^ cell yield during apheresis collection was performed using a flow cytometer (Beckman Coulter or BD FACS instruments) equipped with four solid-state lasers with excitation wavelengths of 488, 515–545, 562–607, and 650 nm. Evaluation of CD34^+^ cell viability was carried out using a trypan blue exclusion test.

If the collection of CD34^+^ HSCs was <0.8 × 10^6^/kg after 2 apheresis sessions or 2 × 10^6^/kg after 4 apheresis sessions, an open-label rescue procedure with G-CSF plus YF-H-2015005 identical to the treatment plan was performed for those patients who rested at least 7 days. A separate informed consent for rescue therapy was obtained.

Patients who achieved ≥2 × 10^6^/kg CD34^+^ HSCs proceeded to AHSCT within 3 months after the last apheresis. Based on the standard procedures of the local study centers, the conditioning regimens included, but not limited to, CBV (cyclophosphamide, carmustine and etoposide), BEAM (carmustine, etoposide, cytarabine, and melphalan) and BEAC (carmustine, etoposide, cytarabine and cyclophosphamide) regimens. G-CSF was initiated at a dose of 5 μg/kg on day 5 after HSC transfusion in both experimental and placebo groups.

### Efficacy and Safety Assessment

The primary efficacy endpoint was the proportion of patients achieving ≥5 × 10^6^/kg CD34^+^ HSCs within ≤4 apheresis sessions. The secondary efficacy endpoints included the cumulative collected number of CD34^+^ HSCs, proportion of patients procuring ≥2 × 10^6^/kg CD34^+^ HSCs in ≤4 apheresis sessions, time to collect ≥2 × 10^6^/kg CD34^+^ HSCs, time to collect ≥5 × 10^6^/kg CD34^+^ HSCs, time to neutrophil engraftment (the first day when the absolute neutrophil count was ≥1.0 × 10^9^/L for 1 day or ≥0.5 × 10^9^/L for 3 consecutive days), and time to platelet engraftment (the first day when the platelet count was ≥20 × 10^9^/L without a transfusion for 7 consecutive days). The parameters for safety assessment included adverse event (AE), serious AE (SAE) and treatment emergent adverse event (TEAE).

### Statistical Analysis

Sample size was calculated with Power Analysis and Sample Size (version 13.0). Given that there is estimated 30% of placebo-treated patients would achieve the primary efficacy endpoint, a minimum sample size required for this trial was 42 patients per group in order to examine a 30% difference with 80% power. The final sample size was estimated to be 50 patients per group due to the consideration of potential dropouts.

Quantitative data analysis was conducted using the Student's *t*-test or Wilcoxon rank sum test. Categorical data were evaluated by the chi-square test or exact probability test. Grade data were compared using the Wilcoxon rank sum test or Cochran-Mantel-Haenszel mean score test. All statistical analyses were performed with Statistical Analysis Software (version 9.4).

## Results

### Patient Characteristics

From January 9, 2017 to December 18, 2018, 101 patients at 15 sites in China were recruited. All patients were randomized into YF-H-2015005 (*n* = 51) and placebo (*n* = 50) groups at a ratio of 1:1. The median age of the patients was 47 years, with a gender ratio of 1:1, for the whole cohort. The most common historical types were diffuse large B-cell lymphoma (*n* = 56) and peripheral T/NK cell lymphoma (*n* = 17). The most common chemotherapy regimens were R-CHOP (rituximab, cyclophosphamide, doxorubicin, vincristine, prednisone) regimen (*n* = 19, 31.7%) in 60 patients treated with front-line chemotherapy and DICE (dexamethasone, ifosfamide, cisplatin, etoposide) regimen (*n* = 17, 41.5%) in 41 patients treated with salvage chemotherapy. As presented in Table 1, the baseline characteristics were relatively similar between the two groups, except for younger median age in YF-H-2015005 group.

**Table 1:** Baseline characteristics.

	**YF-H-2015005 group** **(*n =* 51, %)**	**Placebo group** **(*n =* 50, %)**	***P***
Age, years			0.018
Median	45	50	
Range	18–65	18–64	
Ethnicity			1.000
Han	49 (96.1)	49 (98.0)	
Others	2 (3.9)	1 (2.0)	
Gender			0.767
Male	27 (52.9)	25 (50.0)	
Female	24 (47.1)	25 (50.0)	
Body weight, kg			0.486
Median	63.0	64.5	
Range	46.0–107.0	48.0–97.0	
Pathology type			0.875
Lymphoblastic lymphoma	7 (13.7)	5 (10.0)	
Mature B-cell lymphoma	36 (70.6)	36 (72.0)	
DLBCL	32 (62.7)	24 (48.0)	
FL	0 (0)	2 (4.0)	
MCL	3 (5.9)	3 (6.0)	
MZL	1 (2.0)	1 (2.0)	
SLL	0 (0)	1 (2.0)	
TL	0 (0)	2 (4.0)	
Others	0 (0)	3 (6.0)	
Peripheral T/NK cell lymphoma	8 (15.7)	9 (18.0)	
PTCL NOS	1 (2.0)	3 (6.0)	
ALCL	1 (2.0)	1 (2.0)	
AITL	2 (3.9)	2 (4.0)	
ENKTCL	0 (0)	1 (2.0)	
EATL	1 (2.0)	0 (0)	
SPTCL	2 (3.9)	1 (2.0)	
PCACTL	1 (2.0)	1 (2.0)	
ECOG performance status score			0.205
0	47 (92.2)	42 (84.0)	
1	4 (7.8)	8 (16.0)	
Chemotherapy lines and regimens			0.273
Front-line therapy	33 (64.7)	27 (54)	
R-CHOP	12 (23.5)	7 (14)	
R-EPOCH	2 (3.9)	1 (2)	
CHOP	2 (3.9)	1 (2)	
CHOEP	2 (3.9)	2 (4)	
EPOCH	2 (3.9)	0 (0)	
Others	13 (25.5)	16 (32)	
Salvage therapy	18 (35.3)	23 (46)	
DICE	7 (13.7)	10 (20)	
DHAP	0 (0)	4 (8)	
GEMOX	0 (0)	3 (6)	
Others	11 (21.6)	6 (12)	
Radiotherapy			1.000
Yes	2 (3.9)	1 (2.0)	
No	49 (96.1)	49 (98.0)	
Disease status			0.470
Complete remission	38 (74.5)	34 (68.0)	
Partial remission	13 (25.5)	16 (32.0)	

*AITL, angioimmunoblastic T-cell lymphoma; ALCL, anaplastic large cell lymphoma; CHOEP, cyclophosphamide, doxorubicin, vincristine, etoposide, prednisone; CHOP, cyclophosphamide, doxorubicin, vincristine, prednisone; DHAP, dexamethasone, cytarabine, cisplatin; DICE, dexamethasone, ifosfamide, cisplatin, etoposide; DLBCL, diffuse large B-cell lymphoma; EATL, enteropathy-associated T-cell lymphoma; ECOG, Eastern Cooperative Oncology Group; ENKTCL, extranodal NK/T-cell lymphoma; EPOCH, etoposide, prednisone, vincristine, cyclophosphamide, doxorubicin; FL, follicular lymphoma; MCL, mantle cell lymphoma; GEMOX, gemcitabine, oxaliplatin; MZL, marginal zone lymphoma; PCACTL, primary cutaneous CD8^+^ aggressive epidermotropic cytotoxic T-cell lymphoma; PTCL NOS, peripheral T-cell lymphoma, not otherwise specified; R-CHOP, rituximab, cyclophosphamide, doxorubicin, vincristine, prednisone; R-EPOCH, rituximab, etoposide, prednisone, vincristine, cyclophosphamide, doxorubicin; SLL, small lymphocytic lymphoma; SPTCL, Subcutaneous panniculitis-like T-cell lymphoma; TL, transformed lymphoma*.

### Peripheral Blood CD34^+^ Cells

The median peripheral blood CD34^+^ cells on day 4 between YF-H-2015005 and placebo groups were similar (8.5 vs. 6.0/μL, *P* = 0.119). On day 5, the median peripheral blood CD34^+^ cells increased to 38.0/μL in YF-H-2015005 group and to 9.0/μL in placebo group *P* < 0.001). Moreover, YF-H-2015005 led to 3.1-, 3.1- and 2.4-fold increase of peripheral blood CD34+ cells on day 5 for those patients with threshold values of <10, 10–20, and >20/μL peripheral blood CD34^+^ cells on day 4, respectively (Table 2).

**Table 2:** Comparison of CD34^+^ hematopoietic stem cell yields between the two groups.

	**PB CD34**^****+****^ **count on day 4**					
	** <10 cells/μL**		**10–20 cells/μL**		**>20 cells/μL**	
	**YF-H-2015005** **(*n =* 27)**	**Placebo** **(*n =* 33)**	**YF-H-2015005** **(*n =* 11)**	**Placebo** **(*n =* 11)**	**YF-H-2015005** **(*n =* 13)**	**Placebo** **(*n =* 6)**
**Median PB CD34**^**+**^ **cells count (cells/μL, range)**						
Day 4	7.0 (1.0–9.0)	4.0 (0.0–9.0)	12.0 (10.0–19.0)	12.0 (10.0–20.0)	33.6 (21.0–76.0)	58.0 (29.6–120.0)
Day 5	22.0 (3.0–91.0)	5.0 (1.0–30.0)	37.0 (14.0–77.0)	19.0 (11.0–38.0)	80.0 (30.0–155.0)	74.9 (26.0–198.0)
**Median collected number of HSCs (×10**^**6**^**/kg, range)**						
Day 1	1.6 (0.2–11.4)	0.3 (0.1–2.2)	2.3 (1.1–6.0)	1.1 (0.6–3.7)	6.1 (0.9–10.1)	3.2 (0.9–13.4)
≤4 days	4.3 (0.4–11.4)	0.6 (0.2–6.0)	5.2 (2.4–6.0)	2.3 (1.5–6.8)	6.2 (3.4–10.1)	5.8 (2.0–13.4)
**Number of reaching efficacy endpoint in ≤4 days (%)**						
HSCs ≥ 5 × 10^6^/kg	12 (44.4)	2 (6.1)	8 (72.7)	1 (9.1)	12 (92.3)	4 (66.7)
HSCs ≥ 2 × 10^6^/kg	21 (77.8)	7 (21.2)	10 (100.0)	8 (72.7)	13 (100)	6 (100)

*HSCs, hematopoietic stem cells; PB, peripheral blood*.

### Efficacy

As shown in Table 3, the proportion of patients achieving primary efficacy endpoint was remarkably higher in YF-H-2015005 group than in placebo group (56.9 vs. 12.0%, *P* < 0.001). With respect to secondary efficacy endpoint, the cumulative collected number of CD34^+^ HSCs was greater in YF-H-2015005 (median, 5.10 × 10^6^ cells/kg; range, 0.27–11.12 × 10^6^ cells/kg) than in placebo group (median, 1.67 × 10^6^/kg; range, 0.17–8.09 × 10^6^ cells/kg). YF-H-2015005 increased the proportion of patients mobilizing ≥2 × 10^6^/kg CD34^+^ HSCs within four apheresis sessions (86.3 vs. 38.0%, *P* < 0.001), and shorten the median time to collect ≥2 × 10^6^/kg CD34^+^ HSCs (1 vs. 4 days) or ≥5 × 10^6^/kg CD34^+^ HSCs (3 days vs. not reached). YF-H-2015005 resulted in a significantly higher probability of achieving ≥2 × 10^6^/kg CD34^+^ HSCs or ≥5 × 10^6^/kg CD34^+^ HSCs within 4 apheresis days, especially for those patients who had a threshold of <10 or 10–20/μL peripheral blood CD34^+^ cells (Table 3). Thirty patients (YF-H-2015005, *n* = 4; placebo, *n* = 26) proceeded to rescue therapy, of whom 20 (YF-H-2015005, *n* = 2; placebo, *n* = 18) achieved CD34^+^ HSC collection of ≥2 × 10^6^/kg during rescue therapy.

**Table 3:** Summary of efficacy endpoints.

	**YF-H-2015005 group** **(*n =* 51, %)**	**Placebo group** **(*n =* 50, %)**	***P***
**Primary endpoint**			
≥5 × 10^6^/kg CD34^+^ HSCs within 4 apheresis days	29 (56.9)	6 (12.0)	<0.001
**Secondary endpoint**			
Median cumulative collected number of CD34^+^ HSCs (×10^6^ cells/kg, range)	5.10 (0.27–11.12)	1.67 (0.17–8.09)	<0.001
≥2 × 10^6^/kg CD34^+^ HSCs within 4 apheresis days	44 (86.3)	19 (38.0)	<0.001
Median time to collect ≥2 × 10^6^/kg CD34^+^ HSCs (days)	1	4	<0.001
Median time to collect ≥5 × 10^6^/kg CD34^+^ HSCs (days)	3	Not reached	<0.001
**≥5 × 10**^**6**^**/kg CD34**^**+**^ **HSCs by apheresis day**			
Day 1	15 (29.4)	1 (2.0)	<0.001
Day 2	25 (49.0)	4 (8.0)	<0.001
Day 3	29 (56.9)	5 (10.0)	<0.001
Day 4	29 (56.9)	6 (12.0)	<0.001
**≥2 × 10**^**6**^**/kg CD34**^**+**^ **HSCs by apheresis day**			
Day 1	29 (56.8)	6 (12.0)	<0.001
Day 2	38 (74.5)	15 (30.0)	<0.001
Day 3	42 (82.4)	17 (34.0)	<0.001
Day 4	44 (86.3)	19 (38.0)	<0.001
Median time to neutrophil engraftment (days)	11	11	0.604
Median time to platelet engraftment (days)	13	13	0.830

*HSCs, hematopoietic stem cells*.

Thirty-three patients did not proceed to AHSCT (16 due to disease progression, 11 due to inadequate HSCs, and 6 due to patient's refusal). Finally, 37 patients treated with YF-H-2015005 and 31 patients treated with placebo underwent AHSCT. All patients in YF-H-2015005 group and 94.1% of patients in placebo group had achieved neutrophil engraftment, and the median time to neutrophil recovery was similar between the two groups (11 vs. 11 days). Besides, 94.3% of patients treated with YF-H-2015005 and 88.2% of patients treated with placebo achieved platelet engraftment. The median time to platelet recovery was similar between the two groups (13 vs. 13 days).

### Safety

Overall, the incidence rates of AEs were comparable between YF-H-2015005 and placebo groups. During the mobilization period, 45 (88.2%) patients in YF-H-2015005 group and 49 (98.0%) patients in placebo group suffered from at least one AE, ranging from mild to moderate grades (Table 4). The incidence of TEAE were 39.2 and 34.0% in YF-H-2015005 and placebo groups, respectively (*P* > 0.05). The most common TEAE were diarrhea (14%), elevated alkaline phosphatase (6%), and hyperhidrosis (6%) in YF-H-2015005 group, while hyperuricemia (8%), elevated alkaline phosphatase (8%), and diarrhea (6%) in placebo group.

**Table 4:** Treatment emergent adverse events during HSC mobilization period.

	**YF-H-2015005 group** **(*n* = 51, %)**		**Placebo group** **(*n* = 50, %)**	
	**Any grade**	**Grade ≥ 3**	**Any grade**	**Grade ≥ 3**
Diarrhea	7 (13.7)	0 (0.0)	3 (6.0)	0 (0.0)
Elevated alkaline phosphatase	3 (5.9)	0 (0.0)	4 (8.0)	0 (0.0)
Hyperhidrosis	3 (5.9)	0 (0.0)	0 (0.0)	0 (0.0)
Hypoglycemia	3 (5.9)	3 (5.9)	1 (2.0)	1 (2.0)
Elevated aspartate aminotransferase	2 (3.9)	0 (0.0)	2 (4.0)	0 (0.0)
Elevated lactate dehydrogenase	2 (3.9)	0 (0.0)	3 (6.0)	0 (0.0)
Hypoesthesia	2 (3.9)	0 (0.0)	0 (0.0)	0 (0.0)
Nausea	2 (3.9)	0 (0.0)	1 (2.0)	0 (0.0)
Hypokalemia	1 (2.0)	1 (2.0)	1 (2.0)	0 (0.0)
Elevated bilirubin	1 (2.0)	0 (0.0)	0 (0.0)	0 (0.0)
Thrombocytopenia	1 (2.0)	1 (2.0)	1 (2.0)	0 (0.0)
Dyspnea	1 (2.0)	0 (0.0)	0 (0.0)	0 (0.0)
Cough	1 (2.0)	0 (0.0)	0 (0.0)	0 (0.0)
Insomnia	1 (2.0)	0 (0.0)	0 (0.0)	0 (0.0)
Allergic dermatitis	1 (2.0)	0 (0.0)	0 (0.0)	0 (0.0)
Fever	1 (2.0)	0 (0.0)	1 (2.0)	0 (0.0)
Vomiting	1 (2.0)	0 (0.0)	0 (0.0)	0 (0.0)
Abdominal pain	1 (2.0)	0 (0.0)	0 (0.0)	0 (0.0)
Anemia	1 (2.0)	0 (0.0)	2 (4.0)	0 (0.0)
Hyperuricemia	1 (2.0)	1 (2.0)	4 (8.0)	1 (2.0)
Hypoproteinemia	0 (0.0)	0 (0.0)	1 (2.0)	0 (0.0)
Hypercalcemia	0 (0.0)	0 (0.0)	1 (2.0)	0 (0.0)
Leukocytosis	0 (0.0)	0 (0.0)	2 (4.0)	0 (0.0)
Backache	0 (0.0)	0 (0.0)	1 (2.0)	0 (0.0)
Elevated alanine aminotransferase	0 (0.0)	0 (0.0)	3 (6.0)	0 (0.0)
Edema	0 (0.0)	0 (0.0)	1 (2.0)	0 (0.0)

During the follow-up period, the incidence rates of AE and TEAE were 49.0% (*n* = 25) and 2.0% (*n* = 1) in YF-H-2015005 group, and 52.0% (*n* = 26) and 6.0% (*n* = 3) in placebo group, respectively. Only 1 patient in YF-H-2015005 group experienced TEAE with a manifestation of mild thrombocytopenia. During AHSCT, the incidence rates of febrile neutropenia, infection and intravenous antibiotics were 60.5% (*n* = 23), 34.2% (*n* = 13), and 60.5% (*n* = 23) in YF-H-2015005 group, and 40.0% (*n* = 12), 23.3% (*n* = 7), and 76.7% (*n* = 23) in placebo group, respectively. The median transfusion number of packed red blood cell units and platelet units were 2 and 1 in the two groups. In addition, no severe TEAEs were observed in the two groups during the entire study period.

## Discussion

For patients with NHL, the infused dose of HSCs has an important impact on engraftment kinetics. Although the HSC dose of 2.0 × 10^6^/kg was usually considered as a minimum threshold (14, 15), higher mobilization target with an optimal dose of ≥5.0 × 10^6^/kg CD34^+^ HSCs could improve engraftment (16). In the present study, YF-H-2015005 significantly enhanced the efficacy of HSC mobilization with acceptable toxicity, implying that it can be a potential mobilization strategy in the future.

CXCR4 antagonist has been shown to enhance the HSC mobilization efficacy of G-CSF (17–19). In a randomized study, plerixafor led to 62% of patients reaching the collection target of ≥5.0 × 10^6^/kg CD34^+^ HSCs and 88% achieving the collection target of ≥2.0 × 10^6^/kg CD34^+^ HSCs in Chinese patient population (10). In this clinical trial, the superiority of YF-H-2015005 was comparable to those in the earlier plerixafor trial (10). After treatment with YF-H-2015005, about 57% of patients achieved the optimal mobilization target of ≥5.0 × 10^6^/kg CD34^+^ HSCs, and 86% of patients reached the minimum mobilization target of ≥2.0 × 10^6^/kg CD34^+^ HSCs, which were remarkably higher than those treated with placebo. Apart from that, the combined treatment of YF-H-2015005 plus G-CSF enabled a target collection of 2~5 × 10^6^/kg CD34^+^ HSCs in fewer apheresis sessions. The median time to collect a target of ≥2 × 10^6^/kg CD34^+^ HSCs was reduced to 1 day in patients treated with YF-H-2015-005. These findings indicate that YF-H-2015005 is associated with favorable HSCs mobilization and less medical resource utilization.

Risk-adapted algorithms of plerixafor-based approaches have been developed, in which plerixafor was only used for poor mobilizers. A study (20) involved 136 patients with myeloma or lymphoma with two different plerixafor utilization methods. Between 2012 and 2014, plerixafor was only “just in time” used in 60 patients at high-risk for mobilization failures who had peripheral blood CD34^+^ HSCs <10/μL on day 4, collection yield of <1.0 × 10^6^ CD34+ cells/kg in the first apheresis day, or collection yield of <1.5 × 10^6^ CD34+ cells/kg in the first 2 apheresis days. Compared with the routine use of plerixafor, “just in time” methods could be safe and cost-effective, which saved 40% use of plerixafor and resulted in the similar mobilization success rates. In this study, YF-H-2015005 significantly increased the peripheral blood CD34^+^ cells and improved the successful mobilization rate, especially for those poor mobilizers who had a threshold of <10/μL peripheral blood CD34+ cells. Moreover, rescue therapy with YF-H-2015005 resulted in 69% of patients achieving a minimum CD34^+^ HSC collection of ≥2 × 10^6^/kg, especially in placebo-treated patients who did not obtain adequate CD34+ HSCs during apheresis sessions. These findings highlighted the need of refining the differentiated mobilization strategy with CXCR4 antagonist such as plerixafor or YF-H-2015005, and further study should be warranted, especially in poor HSC mobilizers.

Previous studies reported that the engraftment and post-transplant recovery of NHL patients treated with plerixafor were comparable to those treated with other mobilizing agents (21–23). A prospective study involving 118 patients with Hodgkin lymphoma, multiple myeloma, and NHL showed that the neutrophil and platelet engraftment rates were 95 and 98% and there was no secondary graft failure at 12 months post-transplant (24). In this trial, the time to neutrophil and platelet recovery was similar between the two groups. It should be noted that the incidence rates of AEs and TEAEs observed in YF-H-2015005 group were consistent with those published previously (13). Although AEs were observed in 88.24% of YF-H-2015005-treated patients, most of them were mild to moderate. In addition, no SAEs and treatment-related SAEs were found after treatment with YF-H-2015005 regimen. These findings suggest that YF-H-2015005 is generally well-tolerated, which can be safely used with G-CSF.

## Conclusions

The randomized controlled study demonstrated that the introduction of YF-H-2015005 into G-CSF regimen increased the proportion of NHL patients who met the optimal and minimum target HSCs dose required for AHSCT, and markedly reduced apheresis sessions compared to placebo-treated patients. Therefore, YF-H-2015005 plus G-CSF regimen was deemed as an effective combination with acceptable tolerability for HSC mobilization in NHL patients.

## Data Availability Statement

The raw data supporting the conclusions of this article will be made available by the authors, without undue reservation.

## Ethics Statement

The studies involving human participants were reviewed and approved by Ethics Committee at Peking University Cancer Hospital and Institute and the participating centers' institutional review boards. The patients/participants provided their written informed consent to participate in this study.

## Author Contributions

YSo and JZ designed the study, analyzed the data, and wrote the manuscript. WL, JZ, and YSh contributed to the study concept. SW and YC contributed to study coordination. CY, XY, and MS performed the statistical analysis. All authors contributed to data collection and interpretation, and approved the manuscript.

## Conflict of Interest

SW and FC are employees of Hefei Yifan Biopharmaceuticals Inc. The remaining authors declare that the research was conducted in the absence of any commercial or financial relationships that could be construed as a potential conflict of interest.

## References

[1] Duarte RF Labopin M Bader P Basak GW Bonini C Chabannon C. Indications for haematopoietic stem cell transplantation for haematological diseases, solid tumours and immune disorders: current practice in Europe, 2019. Bone Marrow Transplant. (2019) 54:1525–52. 10.1038/s41409-019-0516-2 30953028

[2] Kanate AS Majhail NS Savani BN Bredeson C Champlin RE Crawford S. Indications for hematopoietic cell transplantation and immune effector cell therapy: guidelines from the American Society for Transplantation and Cellular Therapy. Biol Blood Marrow Transplant. (2020) 26:1247–56. 10.1016/j.bbmt.2020.03.002 32165328

[3] Majhail NS Farnia SH Carpenter PA Champlin RE Crawford S Marks DI. Indications for autologous and allogeneic hematopoietic cell transplantation: guidelines from the American Society for Blood and Marrow Transplantation. Biol Blood Marrow Transplant. (2015) 21:1863–9. 10.1016/j.bbmt.2015.07.032 26256941 PMC4830270

[4] Liu W Ji X Song Y Wang X Zheng W Lin N. Improving survival of 3760 patients with lymphoma: experience of an academic center over two decades. Cancer Med. (2020) 9:3765–74. 10.1002/cam4.3037 32281275 PMC7286476

[5] Gertz MA Wolf RC Micallef IN Gastineau DA. Clinical impact and resource utilization after stem cell mobilization failure in patients with multiple myeloma and lymphoma. Bone Marrow Transplant. (2010) 45:1396–403. 10.1038/bmt.2009.370 20062089

[6] Pusic I Jiang SY Landua S Uy GL Rettig MP Cashen AF. Impact of mobilization and remobilization strategies on achieving sufficient stem cell yields for autologous transplantation. Biol Blood Marrow Transplant. (2008) 14:1045–56. 10.1016/j.bbmt.2008.07.004 18721768

[7] Tekgündüz E Demirkan F Vural F Göker H Özdogu H Kiki I. Current practice of autologous hematopoietic progenitor cell mobilization in adult patients with multiple myeloma and lymphoma: the results of a survey from Turkish hematology research and education group (ThREG). Transfus Apher Sci. (2017) 56:804–8. 10.1016/j.transci.2017.11.010 29153305

[8] Hsu YM Cushing MM. Autologous stem cell mobilization and collection. Hematol Oncol Clin North Am. (2016) 30:573–89. 10.1016/j.hoc.2016.01.004 27112997

[9] DiPersio JF Micallef IN Stiff PJ Bolwell BJ Maziarz RT Jacobsen E. Phase III prospective randomized double-blind placebo-controlled trial of plerixafor plus granulocyte colony-stimulating factor compared with placebo plus granulocyte colony-stimulating factor for autologous stem-cell mobilization and transplantation for patients with non-Hodgkin's lymphoma. J Clin Oncol. (2009) 27:4767–73. 10.1200/JCO.2008.20.7209 19720922

[10] Zhu J Huang H Chen H Zhang X Li Z Wu D. Plerixafor and granulocyte-colony-stimulating factor for mobilization of hematopoietic stem cells for autologous transplantation in Chinese patients with non-Hodgkin's lymphoma: a randomized Phase 3 study. Transfusion. (2018) 58:81–7. 10.1111/trf.14426 29238988

[11] DiPersio JF Stadtmauer EA Nademanee A Micallef IN Stiff PJ Kaufman JL. Plerixafor and G-CSF versus placebo and G-CSF to mobilize hematopoietic stem cells for autologous stem cell transplantation in patients with multiple myeloma. Blood. (2009) 113:5720–6. 10.1182/blood-2008-08-174946 19363221

[12] Brave M Farrell A Ching Lin S Ocheltree T Pope Miksinski S Lee SL. FDA review summary: mozobil in combination with granulocyte colony-stimulating factor to mobilize hematopoietic stem cells to the peripheral blood for collection and subsequent autologous transplantation. Oncology. (2010) 78:282–8. 10.1159/000315736 20530974

[13] Liu W Xie Y Ping L Jiang M Zhang G Cui Y. Safety, efficacy, pharmacokinetic and pharmacodynamic evaluation of YF-H-2015005 for mobilizing hematopoietic stem cells in non-Hodgkin's lymphoma patients. J Cancer. (2020) 11:5635–40. 10.7150/jca.48748 32913458 PMC7477452

[14] Mohty M Hübel K Kröger N Aljurf M Apperley J Basak GW. Autologous haematopoietic stem cell mobilisation in multiple myeloma and lymphoma patients: a position statement from the European Group for Blood and Marrow Transplantation. Bone Marrow Transplant. (2014) 49:865–72. 10.1038/bmt.2014.39 24686988

[15] Duong HK Savani BN Copelan E Devine S Costa LJ Wingard JR. Peripheral blood progenitor cell mobilization for autologous and allogeneic hematopoietic cell transplantation: guidelines from the American Society for Blood and Marrow Transplantation. Biol Blood Marrow Transplant. (2014) 20:1262–73. 10.1016/j.bbmt.2014.05.003 24816581

[16] Giralt S Costa L Schriber J Dipersio J Maziarz R McCarty J. Optimizing autologous stem cell mobilization strategies to improve patient outcomes: consensus guidelines and recommendations. Biol Blood Marrow Transplant. (2014) 20:295–308. 10.1016/j.bbmt.2013.10.013 24141007

[17] Karpova D Ritchey JK Holt MS Abou-Ezzi G Monlish D Batoon L. Continuous blockade of CXCR4 results in dramatic mobilization and expansion of hematopoietic stem and progenitor cells. Blood. (2017) 129:2939–49. 10.1182/blood-2016-10-746909 28400375 PMC5445573

[18] Rettig MP Ansstas G DiPersio JF. Mobilization of hematopoietic stem and progenitor cells using inhibitors of CXCR4 and VLA-4. Leukemia. (2012) 26:34–53. 10.1038/leu.2011.197 21886173 PMC3514440

[19] Larochelle A Krouse A Metzger M Orlic D Donahue RE Fricker S. AMD3100 mobilizes hematopoietic stem cells with long-term repopulating capacity in nonhuman primates. Blood. (2006) 107:3772–8. 10.1182/blood-2005-09-3592 16439684 PMC1895780

[20] Veltri L Cumpston A Shillingburg A Wen S Luo J Leadmon S Hematopoietic progenitor cell mobilization with “just-in-time” plerixafor approach is a cost-effective alternative to routine plerixafor use. Cytotherapy. (2015) 17:1785–92. 10.1016/j.jcyt.2015.09.002 26475754 PMC4700501

[21] Varmavuo V Rimpiläinen J Kuitunen H Nihtinen A Vasala K Mikkola M. Engraftment and outcome after autologous stem cell transplantation in plerixafor-mobilized non-Hodgkin's lymphoma patients. Transfusion. (2014) 54:1243–50. 10.1111/trf.12434 24118008

[22] Valtola J Varmavuo V Ropponen A Nihtinen A Partanen A Vasala K Blood graft cellular composition and posttransplant recovery in non-Hodgkin's lymphoma patients mobilized with or without plerixafor: a prospective comparison. Transfusion. (2015) 55:2358–2368. 10.1111/trf.13170 26018461

[23] Dugan MJ Maziarz RT Bensinger WI Nademanee A Liesveld J Badel K. Safety and preliminary efficacy of plerixafor (Mozobil) in combination with chemotherapy and G-CSF: an open-label, multicenter, exploratory trial in patients with multiple myeloma and non-Hodgkin's lymphoma undergoing stem cell mobilization. Bone Marrow Transplant. (2010) 45:39–47. 10.1038/bmt.2009.119 19483760

[24] Russell N Douglas K Ho AD Nademanee A Liesveld J Badel K. Plerixafor and granulocyte colony-stimulating factor for first-line steady-state autologous peripheral blood stem cell mobilization in lymphoma and multiple myeloma: results of the prospective PREDICT trial. Haematologica. (2013) 98:172–8. 10.3324/haematol.2012.071456 22983579 PMC3561422

